# Emerging Progress of RNA-Based Antitumor Therapeutics

**DOI:** 10.7150/ijbs.83732

**Published:** 2023-06-19

**Authors:** Jiayan Fu, Haiyang Dong, Jian Wu, Yongfeng Jin

**Affiliations:** 1 National Key Laboratory of Advanced Drug Delivery and Release Systems, Zhejiang University, 310058, Hangzhou, China.; 2 MOE Laboratory of Biosystems Homeostasis & Protection, Innovation Center for Cell Signaling Network, College of Life Sciences, Zhejiang University, Hangzhou 310058, China.; 3 Cancer Center, Zhejiang University, 310058, Hangzhou, Zhejiang, China.; 4 Department of Hepatobiliary and Pancreatic Surgery, The First Affiliated Hospital, Zhejiang University School of Medicine, 310006, Hangzhou, Zhejiang, China.; 5 Department of Neurosurgery, The First Affiliated Hospital, School of Medicine, Zhejiang University, 310006, Hangzhou, China.

**Keywords:** RNA, therapeutics, modification, delivery, tumor-treatment

## Abstract

RNA-based therapeutics (e.g., mRNAs, siRNAs, microRNAs, ASOs, and saRNAs) have considerable potential for tumor treatment. The development and optimization of RNA modifications and delivery systems enable the stable and efficient delivery of RNA cargos *in vivo* to elicit an antitumor response. Targeted RNA-based therapeutics with multiple specificities and high efficacies are now available. In this review, we discuss progress in RNA-based antitumor therapeutics, including mRNAs, siRNAs, miRNAs, ASOs, saRNAs, RNA aptamers, and CRISPR-based gene editing. We focus on the immunogenicity, stability, translation efficiency, and delivery of RNA drugs, and summarize their optimization and the development of delivery systems. In addition, we describe the mechanisms by which RNA-based therapeutics induce antitumor responses. Furthermore, we review the merits and limitations of RNA cargos and their therapeutic potential for cancers.

## Introduction

RNA-based therapeutics, including messenger RNAs (mRNAs), small interfering RNAs (siRNAs), microRNAs (miRNAs), antisense oligomers (ASOs), small activating RNAs (saRNAs), RNA aptamers, and CRISPR-based gene editing—have considerable therapeutic potential for genetic diseases 1, 2, 3, 4, 5. Nearly 200 drugs have entered clinical trials for cancer, infectious diseases, autoimmune diseases, and neurodegenerative diseases. Compared to small-molecule and DNA-based drugs, RNA-based therapeutics have several advantages. They can target almost any genetic component and upregulate, downregulate, or abolish the expression of genes encoding a variety of proteins, including those with functions in immunity. In addition, because they are not integrated into the host genome, they have little genotoxicity. Moreover, the high efficiency and controllability of their production facilitate their development.

**Figure 1:**
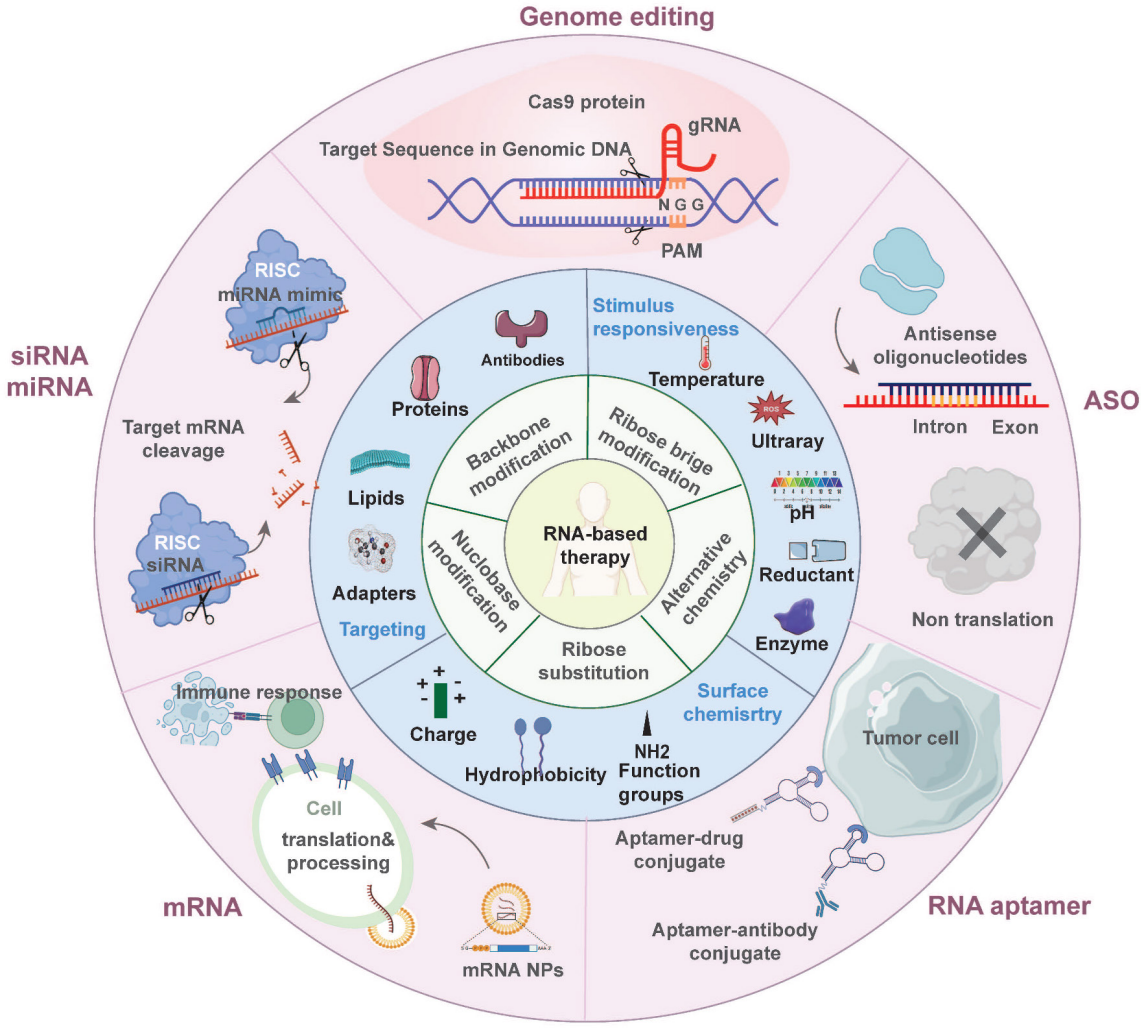
** Overview of RNA-based antitumor therapeutics.** The inner ring discusses the chemical modification strategies of RNA molecules. The middle ring discusses the current optimization for delivery systems. The outer ring introduces various types of RNA-based therapeutics being applied in antitumor treatment.

**Figure 2:**
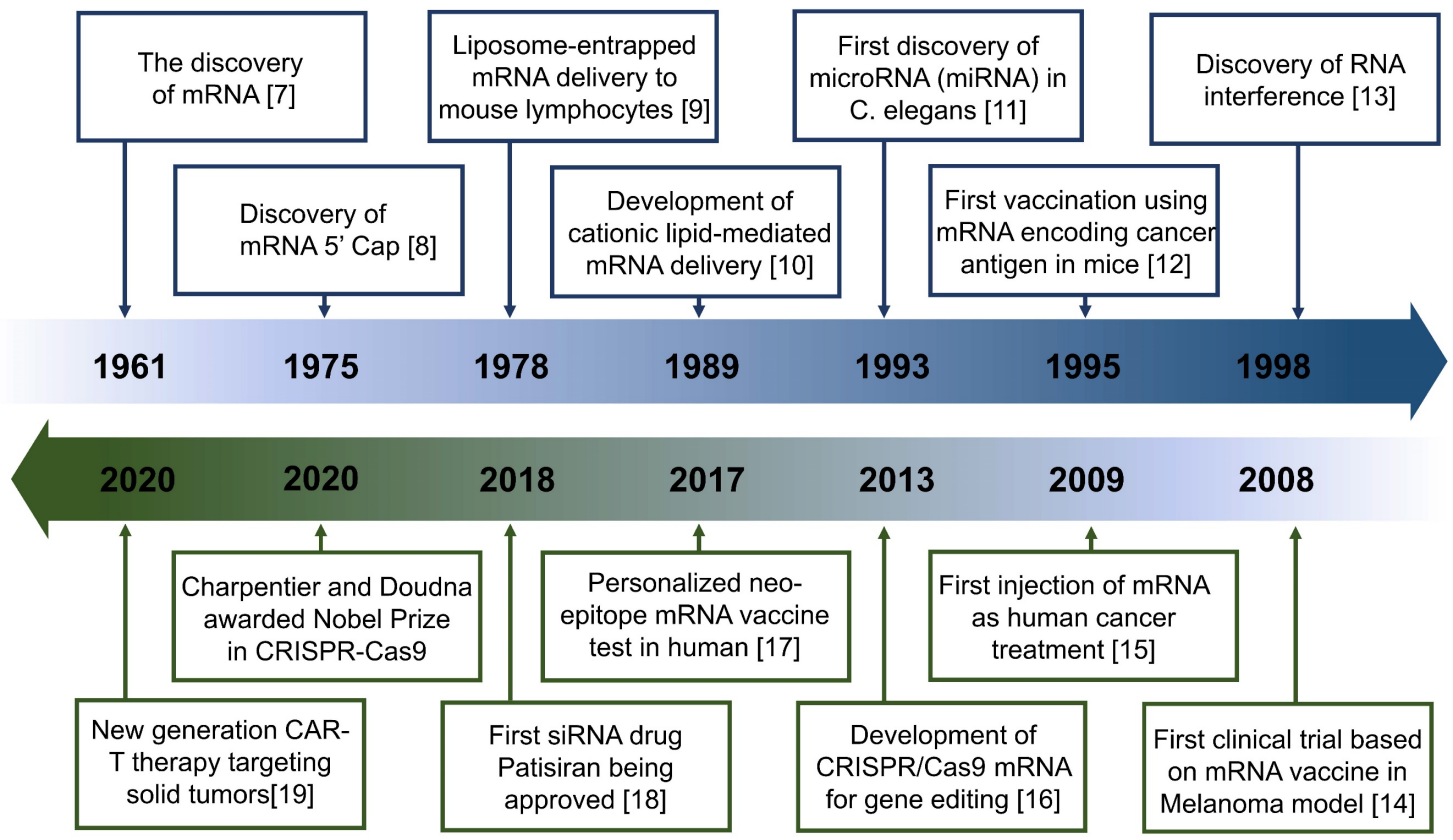
Timeline of major RNA-based therapeutics and development milestones for antitumor treatment.

These advantages are particularly beneficial in tumor treatment. Such therapeutics can control the expression of target proteins, reshape the suppressive tumor microenvironment (TME) by regulating cytokine expression, and induce an innate or adaptive immune response 6 (Figure 1). These mechanisms provide a theoretical basis for the application of antitumor treatment. Indeed, the field of RNA-based therapeutics has expanded considerably in recent years. Several milestone events in RNA-based antitumor treatment are shown in Figure 2 7, 8, 9, 10, 11, 12, 13, 14, 15, 16, 17, 18, 19.

RNA-based therapeutics remains a significant challenge due to the inherent susceptibility of natural RNA to degradation by nucleases. As a result, there is a need to modify RNA structures and optimize delivery vehicles to efficiently deliver nucleic acid cargos to the target tissues and/or organs 20. Chemical modifications can reduce the responses of cellular endogenous immunosensors to double-stranded RNA (dsRNA), greatly improving the safety of these drugs. Enhancing the stability of RNA drugs prevents their degradation by endogenous endonucleases and exonucleases, significantly improving efficacy 21. Nanotechnology-based delivery systems have been a focus of development because viral vectors have genotoxicity and side effects. Nanoparticles (NPs) enable target-specific delivery of therapeutic agents due to their small size, physiological stability, structural tunability, high surface-to-volume ratio, and other favorable characteristics 22, 23. NPs can be engineered to improve their therapeutic effectiveness by attaching cross-linkers and designing stimuli-responsive systems, thereby facilitating their accumulation at target sites and reducing off-target toxicity. Moreover, nanocarriers protect their cargo from degradation in the circulation, thus prolonging half-life.

Here we review recent progress in antitumor RNA-based therapeutics—mRNAs, siRNAs, miRNAs, ASOs, saRNAs, RNA aptamers, and CRISPR-based gene editing. We focus on the mechanisms by which RNA-based therapeutics induce antitumor immune responses. We also discuss RNA modification strategies and classify their carriers according to composition. Moreover, we review the relative merits and bottlenecks of various RNA-based therapeutics in tumor treatment (Figure 1).

## RNA Modification

RNA molecules are inherently unstable. Exogenous RNA molecules trigger immune responses, leading to limited level of protein expression. RNA molecule modifications can be used to address these issues, which is the subject of the next section.

### mRNA modification

mRNA drugs are synthetic versions of mature eukaryotic mRNAs and are typically produced by *in vitro* transcription (IVT). They consist of five main structures: the 5' cap, the 5' and 3' untranslated regions (UTRs), the open reading frames (ORFs) encoding the target proteins, and the 3' poly(A) tail. These structures affect the stability, translation efficiency, and immunogenicity of mRNA-based therapeutics 24.

The 5' and 3' UTRs are non-coding regions whose secondary structures, elements, and lengths affect ribosome recruitment and mRNA translation 25, 26, 27. The 5' UTR directly affects the translation of its downstream ORFs 28. Translated elements of the 5' UTR are referred to as upstream open reading frames (uORFs) 29. For example, the so-called Kozak sequence improves the accuracy of translation initiation by surrounding the start codon with highly conserved nucleotides 30, 31. A completely randomized 10-nucleotide-sequence preceding an uORF would drive translational output and determine mRNA stability, providing insight into the cis-regulatory code in the 5' UTR 32. The complex secondary structure of the GC-rich 5' UTR, such as that of the ornithine decarboxylase mRNA 33, is associated with translation inhibition 27. This explains in part the effect of the 5' UTR on oncogene expression. The presence of additional uAUG motifs in and the complex secondary structure of the 5' UTR prevent translation and suppress BRCA1 expression in breast cancer cells 34. The 3' UTR also contains elements that regulate multiple aspects of mRNA metabolism, such as their nuclear export, cytoplasmic localization, translation efficiency, and stability 35. There is an optimal length for the 3' UTRs: mRNAs with long 3' UTRs have short half-lives, whereas those with short 3' UTRs have low translation efficiency 36. The most commonly used UTR sequences are from those of genes expressed at high levels, e.g., α-globin β-globin 37. Repetitive concatenation of UTR sequences can enhance mRNA stability and translation efficiency 38.

The 5' cap structure contributes to mRNA stability and translation efficiency 1, 39. The biological roles of cap-0 (m7GpppN-), cap-1 (m7GpppNm-), and cap-2 (m7GpppNmNm-) in mRNAs have been widely investigated 40, 41. Uncapped or abnormally capped mRNAs can be recognized by the innate-immune receptor RIG-1, whereas cap-1 and cap-2 prevent recognition by innate-immune sensors 42. Mainstream capping systems include enzymatic capping and the addition of cap analogs co-transcriptionally 43. To date, the capping enzymes from the vaccinia virus are commercially available and widely used for post-transcriptional in vitro capping 44. Under the catalysis of 2'-O-methyltransferase, cap-0 structure can be further modified to form cap-1 or cap-2 cap structures. Enzymatic capping exhibits high capping efficiency but cumbersome production. In contrast, co-transcriptional capping has limited efficiency due to competitive binding to GTP. To prevent reverse incorporation, anti-reverse cap analogs (ARCAs) have been developed to ensure capping only at non-methylated guanosines 45, 46. Co-transcriptional capping using the novel CleanCap™ system does not affect indel formation and has a capping efficiency of 90-99%. The 3' poly(A) tail can also be optimized. Its deletion renders the mRNA molecule unstable 47. Polyadenylation can be engineered by adding a fixed-length poly(A) sequence to the DNA template or enzymatically 1.

Nucleotide modification can also optimize mRNA stability, translation efficiency, and immunogenicity *in vivo*. Activation of Toll-like receptor (TLR) 3 can be inhibited by replacing the original nucleotide with 6-methyladenosine (m^6^A) or 2-thiouridine (s^2^U), whereas replacement with 5-methylcytidine (m^5^C), 5-methyluridine (m^5^U), s^2^U, m^6^A, or pseudouridine (Ψ) blocks the activation of TLR7 and TLR8, thus preventing an innate immune response 21, 48, 49, 50, 51, 52. Indeed, Ψ and m^5^C enhance RNA stability and translational capability while diminishing its immunogenicity 48, 53. N^1^-methyl Ψ-modified nucleotides are employed in IVT to improve the safety and stability of mRNA vaccines. Two mRNA vaccines against severe acute respiratory syndrome coronavirus-2 (SARS-CoV-2) were modified using N^1^-methyl Ψ and each showed > 90% protection against coronavirus disease 2019 (COVID-19) 54. By contrast, another mRNA vaccine candidate failed to reach the expected level of efficacy, potentially due to the lack of nucleotide modification 55.

### siRNA modification

siRNA can be chemically modified to enhance the selectivity of their antisense strands for RNA-induced silencing complex (RISC) loading and to reduce off-target RNAi activity. However, unmodified siRNA may induce immune toxicity by activating TLR3. A variety of chemical modification strategies can be used to maximize the therapeutic efficacy and minimize the side effects of siRNAs.

Chemical modification typically targets the sugar ring, base, and phosphate skeleton of nucleotides. The goals are to improve the binding affinity of siRNA and protect it from nuclease degradation 56. 2'-O-methyl (2'-O-Me) is the most commonly used modification of naturally occurring ribose 57. Modification of 2'-O-methyl to 2'-methoxyethyl (MOE) increases binding affinity and nuclease resistance 58. In addition, 2'-fluorine (2'-F) also improves siRNA binding affinity and is well tolerated due to its similar size and charge 59. 2'-F modified siRNA shows excellent silencing of factor VII gene expression in mouse models.

The most common phosphate skeleton modification is phosphorothioate (PS), which prolongs the half-life in the circulation. Clinically approved siRNA therapeutics (e.g., Lumasiran and Inclisiran) have PS modifications. The phosphorodithioate (PS2) substitution in siRNAs involves the replacement of two non-bridged oxygen atoms. The potency and nuclease resistance of PS2-modified siRNAs are slightly higher than those of PS and unmodified siRNAs. Boron phosphate siRNA may be more effective than phosphorothioate 60. Moreover, 5'-(E)-VP modification of siRNA stabilizes the 5'-end of the guide strand and promotes Ago2 loading, thereby improving tissue retention and gene silencing 61, 62.

Base replacement is another modification strategy for siRNA-based therapeutics. 5'-nitroindole modification of siRNAs reduces passenger strand-mediated off-target effects 63. Due to concern about genome integration of metabolized non-natural residues, base structures present in natural nucleic acids, such as m^5^C and m^6^A, tend to be used 64. Notably, 5-fluoro-2'-deoxyuridine modification of siRNA enhances cytotoxicity 10- to 100-fold and activates multiple apoptotic pathways, which shows therapeutic potential for cancer treatment 65.

N-acetylgalactosamine (GalNAc)-siRNA conjugates enable drug delivery to the liver. Tris-GalNAc binds to asialoglycoprotein receptor, which is highly expressed on the surface of hepatocytes, resulting in rapid endocytosis 66. Several GalNAc-siRNA conjugates have exhibited therapeutic potential in clinical trials 67. In one study, delivery of GalNAc-siRNA by a cholesterol-modified antimicrobial peptide silenced the expression of peptidyl-prolyl cis/trans isomerase (Pin) in a model of orthotopic liver cancer 68, and showed sustainable drug delivery.

### ASO modification

ASOs are susceptible to nuclease degradation 69. Various chemical modification strategies have been explored to increase their efficacy and enzymatic stability and reduce their immunogenicity and off-target toxicity. Similar to siRNAs, 2'-ribose modifications (2'-F, 2'-O-Me, and 2'-O-MOE) can improve binding affinity and resistance to enzymatic degradation. However, caution is needed with 2'-F modifications due to their potential toxicity 70. A prior study showed that ASOs with 2'-F modifications exhibited hepatotoxicity. G-clamp is a cytosine analog that increases ASO binding affinity 71 by forming five hydrogen bonds with complementary guanine nucleobases in the target sequence.

An example of a modified ASO molecule is peptide nucleic acids (PNAs) 72, which have stronger binding affinity to RNA sequences than unmodified ASOs. Cationic engineering 73 and lysine modification 74 have been explored to resolve their low water solubility and poor cellular uptake. Locked nucleic acid (LNA) is a nucleotide derivative containing a disaccharide ring that locks the sugar ring into double ring molecular mode via a methylene bridge. This structure limits the flexibility of the sugar ring. LNA/DNA/RNA pairing products have higher unwinding temperatures and greater biological activities; they also activate RNase H 75. Studies have found that the half-lives of nucleotides with three terminal LNAs are 10-fold longer than unmodified nucleotides 76.

### RNA aptamer modification

The inherent susceptibility of RNA aptamers to nuclease degradation determines their stability 77. Commonly used chemical modifications of the 3'- and 5'-ends, the sugar ring, the phosphodiester backbone, and bases can protect aptamers from degradation and prevent their renal clearance. The strategy of conjugating poly ethylene glycol (PEG)^78^ at the 3'- or 5'- end can partially overcome obstacles such as short serum half-life, high renal clearance rate, and nuclease stability for oligonucleotides. However, long PEG chains reduce their binding affinity and increase their half-life in the circulation 79. Thus, PEG-conjugation involves a trade-off between renal clearance and gene-silencing efficiency. Other terminal modifications can also enhance the stability of RNA aptamers. *In vivo* administration of 5'-cholesterol-modified aptamers significantly prolongs plasma half-life and exposure 80. The most common 3'-end modification is inverted thymidine capping, which promotes RNA-aptamer stability in the circulation 81.

Modifications of the sugar ring can improve nuclease stability and prolong serum half-life. Similarly, the 2'-OH positions of RNA aptamers can be substituted with 2'-F 82, 83, 84, 2'-OMe 85, 2'-NH_2_, 2'-LNA 86, 87, and 2'-d-/l-isonucleoside 88, 89. Substitution of non-natural nucleotides into oligonucleotides is achieved by mutating T7 RNA polymerase. However, such substitutions can lead to nonspecific immune reactions or toxicity. Phosphate-backbone modifications can be introduced to stabilize the phosphodiester bonds, including PS 90 and PS2 91 bonds. The substitution in PS bonds is chiral (*Sp* or *Rp* configuration), unlike the natural conformation, and may therefore have adverse effects on biological function.

### Others

Chemical modification strategies are under development for other RNA-based therapeutics. The nucleotide sugar ring, base, and phosphate skeleton are modified in anti-miRNA oligonucleotides (AMOs). Most widely used chemical modifications on AMOs are LNA, 2'-F-RNA, 2'-OMe, PNAs. Modified AMOs with higher binding affinity and superior stability have greater regulatory potency 92, 93. Oligonucleotides with 2'-F modifications show increased thermal stability (T_m_ +1.6°C, higher than the 2'-OMe modification) 94. The 2'-F modification can be combined with the 2'-MOE substitution to enhance AMO stability *in vivo* 95. Substituting ribonucleotides with LNAs can endow antimir drugs with increased resistance to nuclease degradation and enhanced target affinity 96. This modification strategy has been used for the clinical trial of Miravirsen, an antagomir of miR-122 (NCT01200420). Moreover, adding a non-base modifier to the end of AMO have been reported to increase the T_m_ by mediating hydrophobic stacking interactions 97.

## Delivery Systems for RNA Therapeutics

**Figure 3:**
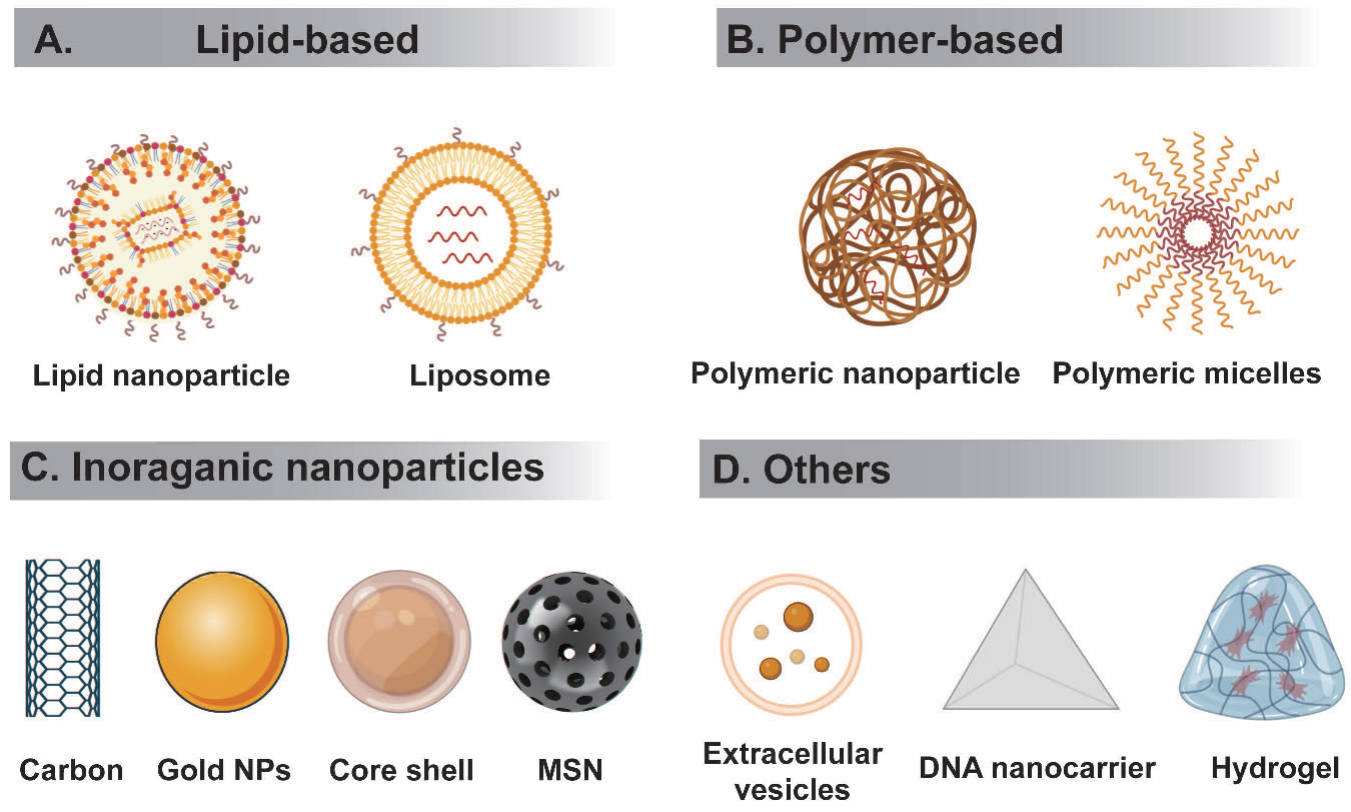
** Schematic representation of different types of nanocarriers used in RNA delivery. (A)** Lipid-based delivery system mainly include liposome and lipid nanoparticle, etc. **(B)** Polymer-based delivery system mainly include polymeric nanoparticle, polymer micelles, etc.** (C)** Inorganic nanodelivery system mainly include carbon, metal NPs, core-shell, MSN, etc.** (D)** Others includes extracellular vesicles, DNA origami, hydrogel, etc.

RNA therapeutics must be delivered to the correct tissues without triggering an immune reaction. However, the high molecular weight and negative charge of RNA hampers their delivery to target sites. RNA delivery systems can be divided to viral or nonviral. Below we summarize several nonviral RNA delivery systems being extensively studied (Figure 3).

### Polymer-based NPs

Polymeric NPs are typically prepared from biocompatible and biodegradable polymers, in which the drug is dissolved, entrapped, encapsulated, or attached to a nanoparticle matrix. Polymers bind nucleic acids to form polymeric complexes at physiological pH, facilitating gene delivery. Polymeric NPs promote electrostatic binding to nucleic acid cargo by interacting with positively charged units. Nucleic acids and polymers can be covalently linked using degradable linkers. The addition of cationic groups such as chitosan to polylactic-co-glycolic acid (PLGA) enables its use for siRNA delivery 98. In addition, polyethyleneimine (PEI) and poly(l-lysine) (PLL) form complexes with RNA via electrostatic interactions. Because unmodified PEI and PLL are not well-tolerated 99, they have been chemically modified to enhance their *in vivo* transfection capability and reduce their toxicity 100. The synthetic bio-reducible polymer poly(beta-amino ester) is synthesized by conjugating amine monomers to diacrylates 101. Polymer-based NPs have low nonspecific toxicity due to degradation by hydrolysis as well as by bio-reduction in the reducing environment of the cytoplasm 102.

### Lipid-based NPs

Lipid-based NPs have high biocompatibility and biosafety, and their production is simple. They include liposomes, micelles, emulsions, and solid lipid NPs (SLNs). LNPs have been evaluated as mainstream delivery systems in multiple preclinical trials.

Because the size, shape, surface charge, and materials of NPs affect their cellular uptake 103, LNPs with different molecular structures have been developed for RNA delivery. Helper lipids, such as DOPE and cholesterol, are critical components of LNPs. The structural characteristics of cholesterol are determinants of their intracellular delivery and the efficiency of gene transfection 104. Study revealed that incorporation of C-24 alkyl phytosterols into LNPs enhances gene transfection. The length of the alkyl tail, flexibility of the sterol ring, and polarity are required to maintain high transfection efficiency 104. There are also some concerns about the use of cationic lipids, for those cationic lipids bearing quaternary ammonium groups have potential cytotoxicity and relatively short blood circulation time stemming from the positive charge, which hindered their clinical translation 105. In contrast, the neutrality of ionizable lipids at physiological pH help reduces the toxicity and, to some extent, increases the circulation half-life of ionizable LNPs 106. The loss of mRNA activity in LNP delivery systems is caused by electrophilic impurities derived from the oxidation of ionizable cationic lipid components and subsequent hydrolysis of the tertiary amine 107. Another report speculated that the highly pro-inflammatory effect of LNP-based systems is likely caused by their ionizable lipid components, the removal of which abolishes visible skin inflammation 108. However, mitigating this toxicity by reducing the charge of cationic lipids seem to be unwise because of the descending level of nucleic acid encapsulation and transfection efficiency of LNP-based systems. Therefore, toxicity, immunity, and therapeutic effectiveness must be balanced.

### Inorganic NPs

Inorganic NPs (gold NPs [AuNPs], silver NPs [AgNPs], carbon nanotubes, mesoporous silica NPs [MSNs] and so on) have a narrow size distribution and a surface chemistry suitable for ligand conjugation 109. The physicochemical properties of inorganic NPs are not susceptible to the environment, which makes them suitable for photothermal or photodynamic therapy of solid tumors 110.

AuNPs typically have diameters of 1 to 100 nm, a large surface-to-volume ratio, good optoelectronic properties, excellent biocompatibility, and low toxicity 111. Modifications of the shape, diameter, PEGylation, and surface charge of AuNPs affect their drug-loading capacities 110. Due to their unique properties, AuNPs have been applied in conjunction with chemotherapy or photothermal therapy for cancer 112. Currently, various optimization strategies have been developed *in vivo* for extending the plasma half-lives of AuNPs and enhance their targeted accumulation and controllable release 113. Similarly, AgNPs are NPs 1-100 nm in diameter composed of silver atoms 114. In addition to their antibacterial properties, L-cysteine AgNPs have potential for drug delivery and excellent biocompatibility 115.

MSNs are biodegradable and chemically stable nanostructured materials composed of silica particles with pore channels 116. The tunable pore size and mesoporous structure of MSNs facilitate drug dissolution and encapsulation. Furthermore, they exhibit high chemical, thermal, and mechanical stability under physiological conditions, across broad ranges of pH and temperature. Exterior and interior surface modifications of MSNs can improve their therapeutic efficacy and pharmacokinetics. For example, structure-optimized silica nanocarriers coloaded with a TLR9 agonist and antigen exhibit increased accumulation in draining lymph nodes, thereby enhancing antigen-specific B- and T-cell immunity in a murine tumor model 117.

### Others

Versatile multifunctional nanomaterials whose synthesis is simple and inexpensive, as well as hybrids that integrate the advantages of different materials, have been developed 118. An example is exosomes, a type of extracellular vesicle (EV) secreted by most types of cells 119. Exosomes mediate intercellular communication and have functional and structural similarities with synthetic drug carriers such as liposomes 120, thus can serve as potent candidates for drug delivery 121, 122, 123, 124. Exosomes could be used as biomimetic nano-vehicles for gene therapy. The synthesis of DNA nanostructure-based carriers is simple, and they have excellent biocompatibility. DNA nanostructure-based carriers are fabricated 125 as tetrahedrons 126, prisms 127, nanotubes 128, and planar origami 129, 130.

Hydrogels consist of a three-dimensional network swollen with water 131. Compared to directly delivering naked RNA using hydrogels, loading RNA into nanocarriers encapsulated in the hydrogel network improves RNA stability with no need for chemical modifications. In one study, local delivery of mRNA using a chitosan-alginate gel scaffolded lipoplex promoted T-cell proliferation and antibody secretion 132. Similar results were obtained for a COVID-19 subunit vaccine containing CpG/Alum as adjuvants. Injectable polymeric NP-based hydrogels provide broad protection against SARS-CoV-2 variants 133. Hydrogels can deliver RNA cargos packaged in polymers 134, 135 or inorganic NPs 136, 137. Controlled continuous RNA release can be achieved by adjusting the cross-linking density, hydrophilicity, pore size, and other parameters of hydrogels. Modified hydrogels can prolong the retention of antitumor drugs in the tumor tissue 138, thereby enhancing their uptake by cancer cells and reducing their toxic effects on nontarget cells 131, 139. Optimization of their degradability, clearance rate, and controlled release will make hydrogel-based systems more suitable for *in vivo* delivery of RNA-based therapeutics in clinical applications.

## RNA-based Tumor Therapeutics

### mRNA

#### mRNA vaccines

**Figure 4:**
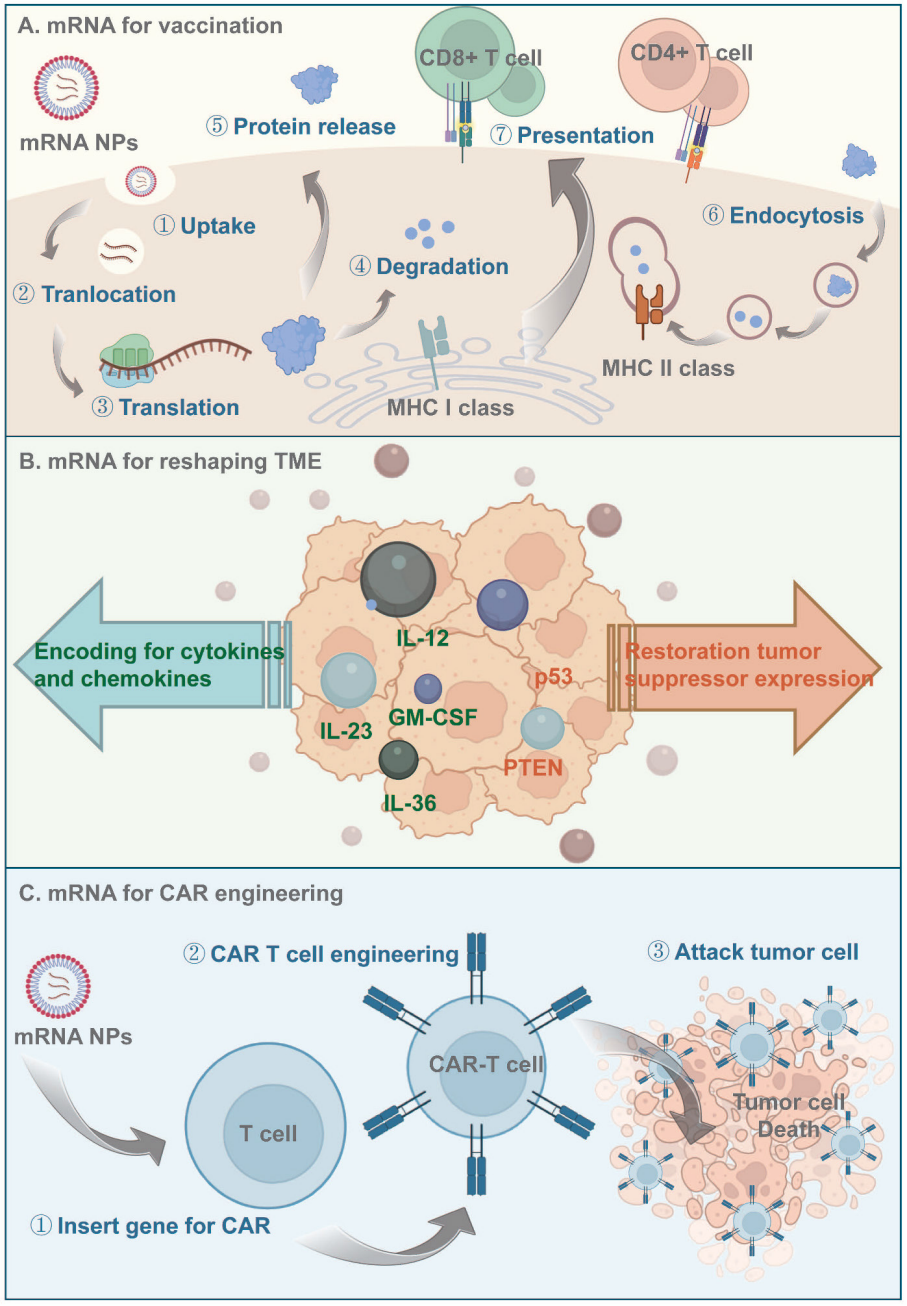
** Principles of mRNA-based antitumor therapeutics. (A)** Antigen-encoding mRNA-based nanoparticles enter the cytoplasm through endocytosis and then translated to protein with the help of ribosome. Those antigen proteins are degraded to peptides by the proteasome and further presented to the APCs via MHC processing. **(B)** mRNA-based nanoparticles being delivered could be translated to proinflammatory cytokines and chemokines to activate the immune signal pathway downstream, they could also reshape the TME by restoring tumor suppressor expression. **(C)** Nanocarriers help effectively deliver CAR-encoding mRNA to the T cell, induce T cell activation and subsequently lead to antigen-specific recognition and tumor tissue elimination.

mRNA-based therapeutics are promising alternatives to DNA for cancer immunotherapy due to their lower mutagenicity and easier transient expression. Furthermore, the *in vitro* production and purification of mRNAs prevents host protein and virus contamination 3, 140. When delivered to antigen-presenting cells (APCs), mRNAs encoding tumor antigens escape to the cytoplasm, where they are translated and processed into peptide epitopes. Subsequently, those peptides bind major histocompatibility complex (MHC) class I and are transferred to the APC surface, activating CD8 T cells and inducing antitumor immune responses 140 (Figure 4). However, the large size, structural instability, and negative charge of naked mRNAs hinder their ability to reach target sites. LNPs are self-assembled nanocarriers that prevent *in vivo* degradation and promote the intracellular delivery and endosomal escape of mRNAs 141. Nucleoside-modified mRNA-LNPs were used by Pfizer/BioNTech and Moderna in their COVID-19 mRNA vaccines 142, 143, and lipid-based systems have subsequently been a focus of interest. Other mRNA LNP formulations have been widely evaluated in preclinical and clinical trials for cancer 144, 145.

LNPs consist of phospholipids, cholesterol, PEGylated lipids, and cationic or ionizable lipids. Phospholipids and cholesterol mediate LNP endocytosis and accelerate mRNA release during endocytosis. PEGylated lipids prolong the half-life in the circulation 146 and act as a steric barrier, preventing aggregation during storage. Thus, the particles could be controlled to an appropriate size. Cationic/ionizable lipids, which serve as the core component of LNPs 147, facilitate binding to negatively charged mRNA molecules and promote their transfer from the endosome to the cytosol for translation via pH-triggered electrostatic interactions with the anionic endosomal membrane 147.

Acute inflammatory responses, such as pain, swelling, and fever, can be caused by mRNA-LNP vaccines 148, 149, 150, 151. These effects are associated with the pro-inflammatory properties of LNPs, and may provide a basis for their adjuvant properties. Previous preclinical data suggested mRNA/LNP complexes show adjuvant activity 152, whereas mRNAs undergo nucleoside modification to attenuate the activation of innate inflammatory pathways. Several cationic/ionizable lipids can induce inflammation by activating TLR pathways 153, 154. In preclinical research on nucleoside-modified mRNA vaccines, LNPs induced considerable neutrophil infiltration and production of inflammatory cytokines and chemokines in mice, independent of the administration route 108. Though ionizable lipids may overcome the pro-inflammatory and cytotoxic effects caused by permanently charged cationic lipids 155. LNP formulations containing ionizable lipids still elicit an innate immune response by releasing IL-l, triggering the secretion of the pro-inflammatory cytokine IL-6 156. The ionizable lipid SM-102 induces inflammasome activation. In addition, PEG, a component of LNPs, is reported to be immunogenic. Repeated administration led those pre-existing PEG antibodies induce complement activation-related pseudo-allergic (CARPA) reactions 157. Therefore, booster shots induce severe adverse reactions, possibly due to further strengthening of the immune memory against LNPs 158. In this sense, LNPs may function not only as carriers of RNAs but also as adjuvants that trigger innate and adaptive immune responses.

Depending on their charge and composition, LNPs can be broadly classified as cationic LNPs, ionizable LNPs, and lipid calcium phosphate (LCP) NPs. Classical mRNA cancer vaccines target tumor-associated antigens (TAAs) preferentially expressed in malignant cells. For instance, Oberli* et al.* 159 reported a lipid NP formulation loaded with the tumor-associated antigens gp100 and TRP2 for the delivery of mRNA vaccines to induce a cytotoxic CD8 T-cell response. The optimized mRNA formulation overcame self-tolerance and significantly prolong survival in transgenic and aggressive mouse melanoma models. More excitingly, replacement of 1% of the molar composition of PEG in the optimized LNP formulation with lipopolysaccharide enhanced the immune response by activating TLRs. Therefore, further investigation of LNPs combined with adjuvants as mRNA vaccine vectors is warranted. However, adding LPS renders manufacture difficult and there is a risk of toxicity. To possibly avoid systemic toxicity, a minimalist vaccine uses heterocyclic lipids as mRNA carriers and self-adjuvants, thereby triggering a stimulator of interferon genes (STING)-mediated type I interferon innate immune response 160. The 12-C-tailed C1 LNP stimulates the expression of inflammatory cytokines such as IL-12 via the TLR4 signaling pathway 161. Cationic lipid-assisted NPs (CLAN) fabricated with biocompatible and biodegradable block copolymer poly (ethylene glycol)-block-poly (lactic-co-glycolic acid) (PEG-b-PLGA) and cationic lipid have been developed for mRNA delivery. They have been used to package OVA-encoded mRNA in dendritic cells *in vitro*, enhancing CD11c-cell maturation and CD8 T-cell proliferation in aggressive E·G7-OVA lymphoma models 162. To further improve intracellular mRNA delivery and the immune response, liposomes modified with a novel cationic and hydrophilic antimicrobial peptide, DP7-C, have been developed. As an immune adjuvant, DP7-C promotes DC maturation and enhances the immune response by stimulating the TLR2-MyD88-IKK-IκB-NF-κB signaling pathway. The carrier and immunoadjuvant functions of the system increase the antitumor effect of a neoantigen-based mRNA vaccine 163.

Several ionizable lipoplex-type mRNA carriers are available. Tateshita* et al.* 164 combined the ssPalmE and KALA peptides to modify NPs for DC-based cancer immunotherapy. Their amphiphilic material consists of a series of ionizable lipids and an SS-cleavable and pH-activated lipid-like material (ssPalm). ssPalm enables cytoplasmic delivery of loaded nucleic acids, and the α-helical cationic KALA-peptide synergistically increases mRNA adjuvant activity by triggering the cytoplasmic nucleic acid sensor. LCP NPs have been used for mRNA delivery in cancer immunology, e.g., LCP for the codelivery of an mRNA encoding a melanoma-associated antigen (TRP2) and an immune checkpoint-targeting siRNA to DCs *in vivo* 165. The calcium phosphate core promotes acid-mediated dissolution in the endolysosomal compartment, triggering rapid cargo release after cellular internalization. The codelivery of a PD-L1 siRNA and an mRNA vaccine elicits a robust and durable antigen-specific immune response in the melanoma model. More stringent requirements have been proposed for delivery-system modification. To overcome the need for repeated administration of NP-based vaccines, in one study, graphene oxide (GO) and low-molecular-weight polyethyleneimine (LPEI) were mixed and used to fabricate an injectable GO-LPEI hydrogel. Use of this injectable hydrogel nanocarrier to deliver OVA-encoding mRNA generated ovalbumin and adjuvant-laden nanovaccines and markedly increased the number of antigen-specific CD8 T cells, thereby inhibiting tumor growth 166.

The clinical applications of the above delivery system are hampered by the lack of specific targeting, which make them useful only for local inoculation and liver-targeted therapy. Efficient lymphatic drainage and accumulation can be promoted by PEGylation and modifying NP size and surface charge 167 of LNP-based mRNA therapeutics. Xu* et al.* 168 found that the delivery and targeting of LNPs are modulated by the head chemical structure. They screened an endogenously LN-targeting lipid NP, 113-O12B. Compared to Pfizer/BioNTech's mRNA vaccine, ALC-0315, 113-O12B targeted lymph nodes and the liver at a 3:1 ratio, showing increased lymph-node and significantly decreased liver mRNA expression. Encapsulation of a TRP-2 peptide-encoding mRNA markedly inhibited tumor growth. Moreover, mice with complete remission did not show new tumor formation after injection of metastatic tumor cells, indicating induction of long-term immune memory 168. Kranz* et al.* reported that a decreased cationic lipid-to-DOPE ratio of mRNA-loaded lipoplexes affected organ specificity. Based on this rationale, they developed a lipoplex-based system for cargo delivery to splenic DCs 169. These RNA-LPX complexes showed synchronized induction of highly potent adaptive and type-I-IFN-mediated innate immune responses for cancer immunotherapy.

#### mRNA for reshaping the TME

Use of mRNAs to restore tumor-suppressor expression has therapeutic potential for cancer. An example is a polymeric NP platform for delivering mRNA encoding phosphatase and tensin homolog deleted on chromosome ten (PTEN), a cancer-inhibiting factor. Reactivating PTEN in PTEN-mutated melanoma cells and PTEN-null prostate cancer cells by mRNA delivery reversed the immunosuppressive TME by promoting CD8 T-cell infiltration and lifting the expression level of proinflammatory cytokines 170. The results suggested that this PTEN-NP platform elicited a robust and safe antitumor immune response by inducing tumor-cell autophagy and releasing damage-associated molecular patterns (DAMPs), thereby triggering tumor immunogenic cell death (ICD) and sensitizing cancers to immune checkpoint blockade (ICB) therapy.

p53 is a tumor suppressor involved in cell cycle arrest, apoptosis, senescence, and other cellular pathways 171. Beyond its autonomous tumor-suppressive effect, it regulates the TME by modulating the interactions between tumor cells and immune cells. In one study, a modified lipid-polymer hybrid NP platform for mRNA delivery enhanced the selectivity of CXCR4 targeting. A series of ionizable lipid-like compounds and varying densities of CXCR4-targeting ligands were screened for mRNA translation efficiency and HCC-targeting specificity *in vivo*. The CXCR4-targeting NP system transported p53 mRNA to HCC cells, restoring p53 activity and decreasing HCC cell viability. This mRNA nanotherapy-based p53 restoration strategy in combination with anti-PD-1 therapy induced a potent antitumor effect in intrahepatic and ectopic models of HCC with p53 loss. Therefore, combining p53 mRNA therapeutics with ICB could reverse immunosuppression in HCC 171. The introduction of LCOR mRNA into tumor cells can restore the expression of LCOR, a tumor suppressor, by modulating IFN sensitivity 172. In that study, mice serially administered EV-based LCOR mRNA and anti-PD-L1 therapies in combination showed significantly longer survival and complete elimination of lung metastasis. Therefore, LCOR mRNA delivery in conjunction with ICB has potential for specifically modulating antigen presentation in tumor cells.

mRNAs encoding cytokines or chemokines can also induce APC maturation and activation, activate T-cell-mediated immunity, and adjust the dysfunctional immune TME. The mRNA-based adjuvant TriMix consists of mRNAs encoding the costimulatory molecule CD70, the activation stimulator CD40 ligand (CD40L), and constitutively active TLR4 (caTLR4). Upon codelivery of tumor-associated antigen (TAA), the TriMix mRNA reprograms CD8^+^ TiDCs *in vivo* into stimulatory cells that efficiently process spontaneously engulfed TAAs, upregulate costimulatory molecules, and migrate to TDLNs to activate cytotoxic T lymphocytes (CTLs), ultimately delaying the growth of established tumors 173.

Moderna has collaborated with AstraZeneca to develop a local intratumoral mRNA therapy for IL-12 delivery 174. As a crucial mediator of the Th1 immune response, IL-12 facilitates the activation and cytotoxicity of natural killer (NK) cells and CTLs via the IFN-γ signaling pathway. Intratumoral injection of LNP-formulated mIL12 (MEDI1191) induced tumor regression in superficial and deep-seated lesion models, with upregulation of CD8^+^ T-cell infiltration and IFN-γ expression. The systematic administration of such cytokines leads to high exposure, possibly resulting in toxicity. For this reason, MEDI1191, an optimized IL-12 mRNA, was developed for tumor-targeted local delivery. To minimize off-target liver toxicity, the miRNA-mediated binding site was incorporated into the 3'-UTR of the IL-12 mRNA to promote its elimination 175.

Preclinical data suggest limited antitumor activity for IL-12 mRNA monotherapy. For this reason, other mRNA therapeutics encoding mixtures of cytokines and chemokines have been developed. Intratumoral injection of DAL4-LNP-IL-12 and IL-27 mRNAs showed synergistic suppression of tumor growth with robust infiltration of immune effector cells 176. Another research investigated the optimal combination of different cytokines, and demonstrated that a mixture of mRNAs encoding IL-12 single chain, interferon-α (IFN-α), granulocyte-macrophage colony-stimulating factor (GM-CSF), and IL-15 increased systemic antigen-specific T-cell expansion and granzyme B T-cell infiltration, thereby promoting tumor regression 177. Moderna has announced two other local-injection mRNA therapeutics (mRNA-2416 and mRNA-2572), which encode multiple immunoregulatory factors. mRNA-2416 encodes OX40L, dosed alone or in combination with the intravenously administered PD-L1 inhibitor durvalumab for the treatment of lymphoma and metastatic ovarian cancer (NCT03323398). mRNA-2572 includes OX40L, IL-23, and IL-36*γ* mRNAs and is intended for the treatment of lymphoma (NCT03739931). OX40L enhances the expansion and survival of CD4 and CD8 T cells, and IL-23 and IL-36γ are pro-inflammatory cytokines of the IL-12 and IL-1 families, respectively, which activate and mature DCs and other immune cells. Compared to mono-cytokine mRNA therapy, addition of an mRNA encoding the T-cell costimulator OX40L increased the complete response rates of treated and untreated distal tumors. Mice treated with the mixture exhibited complete immune responses and effective protection 178. In summary, the above multi-cytokine or chemokine strategies elicit durable and robust antitumor protection (Figure 4, Table 1).

#### mRNAs for CAR engineering

Engineered T cells that express chimeric antigen receptors (CARs) for adoptive cell therapy (ACT) have considerable benefits for the treatment of certain blood malignancies 179, 180. CARs are recombinant receptor constructs containing replaceable intracellular T-cell signaling domains, targeting domains, and transmembrane domains, enabling the substitution of antigen-binding domains encoded by single-chain variable fragments (scFv). Thus, different signal transduction pathways activate different T-cell functions and properties. The activation mediated by intra-cytoplasmic signaling domains could promote tumor targeting by inducing the release of granzyme and perforin, as well as facilitating tumor killing via activation of other immune components. Although CAR-T therapy has much therapeutic potential for cancer, challenges remain in terms of “on-target, off-tumor” cytotoxicity and feasibility for individuals with severe immunodeficiency 181, 182.

##### NPs as vehicles for CAR delivery

Clinical-scale manufacturing of engineered T cells requires their isolation, transfection, modification, amplification, and re-injection, which is difficult and costly. Although virus-mediated transfer can prolong transgene expression by T cells 183 and has been used for the transduction of CARs into T cells 184, it is time-consuming and its clinical application is hampered by safety concerns, such as mutagenicity and genome-insertion toxicity. NPs have potential for CAR mRNA delivery and have higher transfection efficiency, lower cost, and fewer off-target effects than viral vectors (Figure 4).

Nanocarrier-mediated targeted delivery of an mRNA encoding a rare-cleaving megaTAL nuclease disrupts T-cell receptor expression 185. Surface-anchored targeting ligands of anti-CD3 and anti-CD8 antibodies mediate selective binding of the NPs to T cells and initiate rapid receptor-induced endocytosis. Polyglutamic acid (PGA)-coated surfaces were designed to minimize off-target binding by shielding surface charges. This lymphocyte-targeted NP system improves the therapeutic activity of CAR-T cells by reprogramming them towards a TCM-like phenotype. mRNA NPs transiently expressing the transcription factor Foxo1, which mediates effector-cell differentiation into functionally competent memory cells, induce persistent changes in surface markers and improved antitumor efficacy.

Ionizable lipid NPs have been used to deliver mRNAs to primary human T cells *ex vivo* 145 and they show equivalent CAR expression but less cytotoxicity than electroporation. Ionizable lipids in ethanol were combined with cholesterol (NP stability and membrane fusion), DOPE (endosomal escape), and C14-PEG (suppresses aggregation and nonspecific endocytosis. In another orthogonal experiment for optimizing lipid nanoparticles, the results also showed the impact of excipient on LNP performance and CAR-T reprogramming efficiency 186.

##### NPs combined with CAR

Most solid malignancies failed to effectively respond to CAR-T cell infusion because of tumor resistance, tumor-antigen-escape relapse, and the suppressive TME 187. On the one hand, the dense tumor tissue and compact extracellular matrix are tightly crosslinked, and the resulting pressure hampers the infiltration of CAR-T cells into tumors 188. On the other hand, the TME is not conducive to CAR-T cell survival because of hypoxia, low levels of nutrients, acidic pH, and high permeability. In addition, a variety of immunosuppressive cells and immune checkpoints (PD1, CTLA4) inhibit the killing activity of CAR-T cells 189. Therefore, NPs, with their intrinsic properties, could improve the anticancer efficacy of CAR-T by enhancing cargo activity and stability, stimulating CAR‐T cell proliferation and survival, and increasing *in vivo* delivery efficiency.

In one study, a liposomal antigen-encoding RNA was intravenously administered to stimulate tumor-associated T cells in patients with cancer 19. This CAR-T cell-amplifying RNA vaccine, referred to as CARVac, induced the expression of CLDN6 on DCs, thereby stimulating cytokine secretion and the proliferation of co-cultured CLDN6 CAR-T cells in a dose-dependent manner. The RNA vaccine completely induced tumor regression in an ovarian cancer model compared to CLDN6 CAR-T therapy alone. This pioneering method led to the development of the next-generation drug BNT211, which targets solid tumors. Preliminary results from a dose-exploration clinical trial showed that RNA vaccine (CARVac) comprising CLDN6 CAR-T cells combined with CAR-T showed good safety and efficacy. After 6 weeks of treatment, 4 of 14 patients with testicular cancer and 2 with ovarian cancer showed partial remission. In addition, the target lesions were reduced in size. One patient had no change compared to pretreatment, and two patients showed progression. The objective remission rate was 43%, and the disease control rate was 86% (NCT04503278).

CAR targeting macrophages rather than T cells has emerged as another meaningful strategy for the treatment of solid tumors. MT-101 is a new class of non-T cell CARs produced by transforming monocytes with mRNAs. Most monocytes in blood differentiate into macrophages after migrating to tissues. MT-101 targets tumor cells in peripheral tissues by expressing CARs targeting CD5 on the surface of monocytes. A phase 1/2 clinical study demonstrated the safety and tolerability of MT-101 in patients with refractory or relapsed peripheral T-cell lymphoma (PTCL) at day 28, with no dose-limiting toxicity, cytokine release syndrome (CRS), or immune effector cell-associated neurotoxicity syndrome (ICANS) (NCT05138458). In 2020, Moderna and Carisma Therapies established a cooperative relationship to combine Carisma's engineered macrophage technology with Moderna's mRNA and LNP technology and launched the first clinical study of their so-called CAR-M therapy (CT-0508). The combination of CT-0508 with an anti-PD-1 antibody (pembrolizumab) was used in the phase 1 clinical development stage to treat solid tumors with HER-2 overexpression.

### siRNA

**Figure 5:**
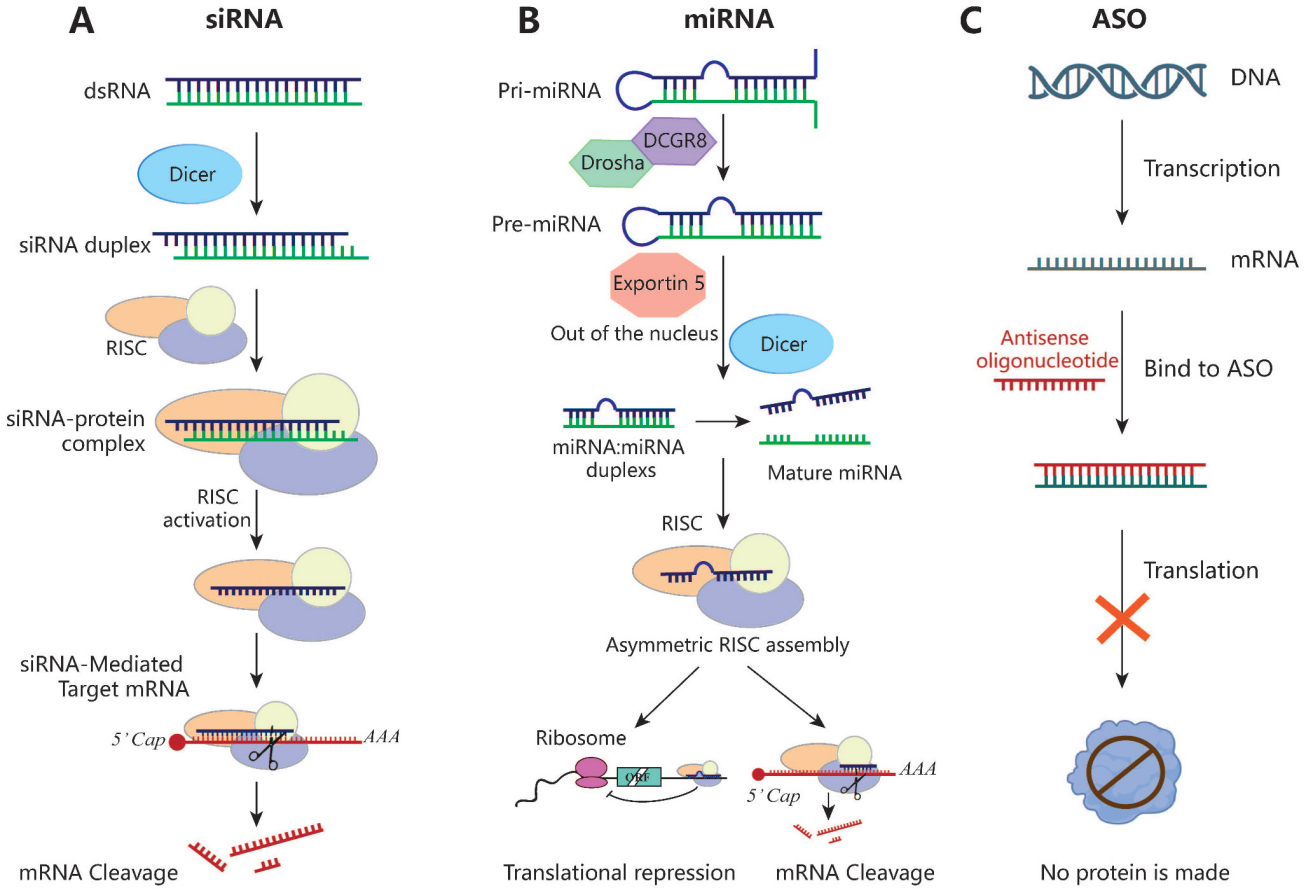
** Schematic of the mechanisms of siRNA, miRNA and ASO. (A)** siRNA inhibits the expression of target genes through Dicer and the RISC, leading to mRNA breakdown and avoiding the expression of the corresponding proteins. **(B)** The pri-miRNA is processed twice to form a mature miRNA. miRNA complementarily binds the 3' UTR of the target gene during transcription or translation and then directly cut the mRNA or directly inhibit the translation process. **(C)** ASO specifically binds to the target mRNA, forming a DNA-RNA hybrid that then triggers mRNA cleavage via RNase H recruitment, ultimately leading to the mRNA level reduction.

siRNAs are double-stranded RNA molecules of 21 to 23 nucleotides, typically with two free bases at the 3'-end, that silence target genes by RNA interference. The precursor is recognized by Dicer RNase and subsequently binds to the target mRNA via the RISC and cleaves it at bases 10 to 11 from the 5'-end, resulting in post-transcriptional gene silencing 190 (Figure 5). Unlike other RNAi technologies, each siRNA can bind to only one mRNA target. Owing to its well-tolerated nature and few side effects, siRNAs have been used to treat various tumors in rodent models 191, 192. However, the development of siRNA-based tumor therapies is hampered by the selection of a suitable targeted delivery method with few systemic side effects.

siRNAs can be used in combination with nanomaterials. Currently, various lipid-based delivery systems have been used for co-delivering siRNA and drugs (Table 2). Cationic liposomes protect the siRNA cargo from enzymatic digestion and prevent its renal clearance. Guo* et al.* 193 used 50 nm cationic lipid-polymer hybrid NPs (LPHs) packed with siRNA in combination with microbubble-enhanced focused ultrasound (MB-FUS) to enhance siRNA delivery in the preclinical brain TME in children and adults by more than 10-fold. In a smoothened (SMO)-activated medulloblastoma model, MB-FUS delivery of SMO-targeted siRNA significantly reduced the production of SMO protein and promoted tumor-cell death 193. In addition, the combination of As_2_O_3_ and HER2-siRNA shows an excellent antitumor effect in an orthotopic gastric tumor model. As_2_O_3_ induces apoptosis and suppresses tumor metastasis, and HER2-siRNA blocks the expression of the oncogene HER2, inhibiting tumor invasion and metastasis 194. The cRGD peptide-modified nanocarrier enabled pH-triggered drug release—the pH-sensitive shell rapidly dissolved in the acidic lysosome, enabling efficient lysosomal escape and release of siRNA to achieve efficient gene silencing 195, 196. A cationic amphiphile containing an endosomal pH-sensitive imidazole ring can be used to deliver both paclitaxel and a Bcl-2 siRNA, significantly inhibiting cellular proliferation and reducing tumor growth 197.

Suitable modification of nanocarriers enables siRNA delivery to target tissues for cancer treatment. One study introduced a versatile codelivery platform for the treatment of triple-negative breast cancer 198. The nanocomplex was modified with hyaluronic acid to specifically target CD44 on TNBC cells. The codelivery of cabazitaxel (a microtubule stabilizer) and IKBKE siRNA (a TNBC oncogene) showed high tumor accumulation and antitumor activity in an orthotopic TNBC mouse model. In addition, integrative hybrid nanoplexes (EhCv/siRNA NPs) prepared from endoplasmic reticulum membranes isolated from cancer cells transported an siRNA via the endosome-Golgi-ER pathway. This method avoids lysosomal degradation and enhances siRNA silencing and antitumor activity against MCF-7 human breast cancer cells in nude mice 199. An NP for dual-targeted immune therapy with a tumor-targeting peptide (SP94) enhances the tumor accumulation of NPs and the intracellular delivery of the therapeutic pDNA/siRNA to HCC cells 200.

Silencing key elements of tumor progression or downregulating immunosuppressive genes can induce an antitumor immune response 201. siRNA-based nanotherapeutics targeting tumor cells downregulate immune-checkpoint proteins (e.g., PD-L1), so-called “don't-eat-me” signals (e.g., CD47), and anti-inflammatory cytokines to induce an antitumor immune response 202. Programmed cell death protein-1 (PD-1) is an immune checkpoint molecule that impairs T-cell activity and induces T-cell depletion. Injection of liposomal nanoparticles loaded with PD-1 siRNA into B16F10 tumor-bearing mice enhances the T cell-mediated antitumor immune response and improves survival 203. PD-1 is also expressed by B cells, macrophages, and NK cells 204. Tumor-associated macrophages (TAMs) overexpressing PD-1 inhibit the phagocytosis by TAMs of PD-L1-expressing tumor cells 205. Hanafy* et al.* 206 reported that PD-1 siRNA encapsulated in SLNs downregulated PD-1 expression in TAMs and mouse tumor tissues and inhibited tumor growth in a mouse model. In another study, delivery by neutral nanoliposomes of mTOR-siRNA to rats with breast cancer enhanced antitumor efficacy by silencing the oncogenic gene mTOR and promoting apoptosis 207.

Research is now focusing on combination therapy or codelivery via nanomaterials with siRNA. Guo* et al.* 208 grafted the photosensitizer pheophorbide A (PPA) onto the DNA backbone at the phosphorothioate modification site and to a PD-1 siRNA linker via supramolecular self-assembly to form a siRNA and PPA co-packaged nanogel. The nanogel photodynamically killed tumor cells, and inhibited PD-L1 expression in tumor cells, thereby synergistically increasing the antitumor immune response. Tumor-targeted lipid-dendrimer-calcium-phosphate NPs with thymosin-functional dendritic polymers have been used to deliver PD-L1 siRNA and immunostimulatory IL-2 encoding plasmid DNA to HCC. They increase tumor infiltration and CD8^+^ T-cell activation, enhancing the effectiveness of cancer immunotherapy and inhibiting HCC progression 200. Selective targeting and reshaping of the immunosuppressive TME by dual delivery of siRNA and plasmid DNA has been attempted to improve cancer immunotherapy 200. In another study, an *in situ*-injectable chitosan hydrogel containing C-C chemokine ligand 5 (CCL5) siRNA-loaded NPs, together with mRNA encoding lipid-immune regulatory factor 5 (IRF5), reshaped the TME in a model of pancreatic cancer by promoting M1 macrophage polarization 209. Delivery of JQ1 (indirect inhibitor of MYC) and a CD47 siRNA in cationic lipid NPs significantly inhibits the expression of PD-L1 and CD47, significantly improving therapeutic efficacy in a mouse model of triple-negative breast cancer 210.

Combination therapy with siRNA and chemotherapeutic drugs has shown promise for some types of cancer. Chen* et al.* 211 reported that chemotherapeutic drugs induce cancer cells to overexpress Xkr8 (a scrambling enzyme activated during apoptosis) at the transcriptional level *in vitro* and *in vivo*. Coadministration of nanocarriers loaded with Xkr8 siRNA and the FuOXP prodrug into tumors via intravenous injection significantly inhibited tumor growth in colon and pancreatic cancer models and enhanced antitumor immune responses. In addition, doxorubicin (Dox) induces ICD in tumors, a type of apoptosis that enhances the protective immune response. Stable nucleic acid-lipid particles (SNALPs) have been used for the simultaneous delivery of Dox and an siRNA that knocks down CD47 (siCD47) 212, the combination therapy synergistically enhances ICD and shows potent antitumor activity 212. Codelivery of Dox and an siRNA using a hydrogel containing an enzyme-cleavable peptide motif overcomes drug resistance by enabling controlled spatiotemporal release 213.

### ASOs

ASOs are synthetic single-strand nucleic acids of 12 to 30 nucleotides. Upon entering a cell, they specifically bind to the target mRNA, forming a DNA-RNA hybrid that triggers mRNA cleavage via recruitment of RNase H, reducing the mRNA level 214. The single strand of ASOs enables targeting and specificity via binding interactions (Figure 5). Because ASOs are based on nucleotide sequences, they can be used to develop protein-associated inhibitors suitable for use in conjunction with traditional therapeutic approaches. Furthermore, they can be chemically modified to improve their stability and resistance to DNases 215. In addition to the 2'-MOE modification, next-generation ASOs have a phosphorothioate (PS) backbone and 2'-4' constrained ethyl (cEt) chemistry at both ends 216, which improve their effectiveness.

Because conventional methods cannot downregulate the protein level of STAT3, Hong* et al.* used a 2.5-generation cEt-ASO (AZD9150) to suppress STAT3 expression, resulting in antitumor activity in lymphoma and lung cancer models 217. In a preclinical study, AZD4785 218, a high-affinity cEt-ASO targeting KRAS mRNA, selectively depleted KRAS mRNA and protein, selectively inhibited the downstream pathways, and suppressed the proliferation of KRAS-mutant cells. AZD4785 targeted KRAS in tumors and showed antitumor activity in mouse and primate models 218.

The limited cell membrane permeability and lack of nuclear targeting of ASOs hinder their clinical application. To address these issues, Cheng* et al.* 219 implemented a gapmer-based design for ASOs by adding 2'-O-methyl modifications with PS linkages, which protected ASOs from nuclease degradation and enhanced their RNase H-mediated cleavage. ASOs targeting both Bcl-2 and Akt-1 were loaded onto lipid NPs to increase their stability. Several studies have evaluated ASO-conjugated nanocarriers for cancer treatment. For instance, the T7 peptide, which has high affinity for the transferrin receptor, was conjugated to a nanocarrier for specific tumor targeting. An ASO-based gene therapy drug for homozygous familial hypercholesterolemia 220 received O-methyl moieties on the terminal ribose groups. The ASO together with a nucleus-targeting TAT peptide packaged in Au NPs shows promise for controlling cancer metastasis 221. Codelivery of ASOs with siRNAs or other drugs may have a synergistic effect. Codelivery siRNA and ASO responds to NIR light 222. Upon NIR light irradiation, the oxygen-cleavable linker between the siRNA and pASO promotes the lysosomal escape of the siRNA and pASO. A multifunctional DNA origami-based nanocarrier for codelivery of doxorubicin and dual-targeted ASOs significantly silences Bcl2 and P-gp and induces apoptosis, enhancing therapeutic effectiveness 130.

### miRNAs

miRNAs are single-stranded, non-coding RNAs typically of 20 to 24 nucleotides. They complementarily bind the 3' UTR of the target mRNA, and then cleave the mRNA or inhibit its translation 223, 224 (Figure 5). miRNAs regulate gene expression via silencing, upregulation, translation activation, or post-transcriptionally. They modulate tumor progression by affecting the interactions between tumor cells and immune cells. miRNA-based therapeutics can restore tumor-suppressor miRNA levels (miRNA mimics and other small-molecule drugs) or block oncomiR function (locked nucleic acids or miRNA sponges) 225. However, naked miRNA mimics are unstable and easily degraded by nucleases, thus requiring a suitable delivery system.

The first clinical miRNA-based therapeutic was the liposomal formulation of miR-34a, named MRX34 226. miR-34a is a tumor-suppressor miRNA that is dysregulated by p53. In phase 1 studies, delivery of miR-34a using liposome restored a normal tumor-suppressor pathway, inducing apoptosis in tumor cells *in vitro* and in mouse models of cancer. In addition, mice treated with MRX34 exhibited significant tumor growth inhibition, and a significantly increased survival rate. However, liposomal delivery systems have issues with liver and kidney accumulation and acute hypersensitivity reactions.

Inorganic NPs are superior to liposomes in terms of their adjustable size and superior pharmacokinetics. For example, a lipid-coated calcium phosphonate NP has been designed for macrophage-targeted miRNA delivery in tumor immunotherapy 227. Mannose conjugation and a pH-responsive steric shielding enable this nanocarrier to reach macrophages and release miRNA-155 in the acidic TME, reactivating protumor TAMs to antitumor macrophages, thereby inhibiting tumor growth without marked off-target effects. Similar TAM-targeting strategies have been used in a non-small cell lung cancer mouse model 228 and epithelial ovarian cancer model 229. HA-based polymeric NPs have been modified to deliver miRNA-125b to CD44 macrophages. The augmented expression of miR-125b markedly inhibits primary tumor growth and repolarizes TAMs 228, suggestive of therapeutic potential.

Vesicular exosomes could be used for miRNA delivery. Exosomes, the smallest extracellular vesicles (EVs) (40 to 160 nm), can transport miRNAs in a paracrine and endocrine manner 230. miRNA-containing exosomes are taken up via receptor-ligand interaction and subsequently regulate gene expression in recipient cells 231. Exosomes can overcome the limitations of liposomes, such as their membrane toxicity and low biocompatibility with target ligands. Their small size promotes the penetration of, and retention in, solid tumors, suggesting potential for tumor immunotherapy. EVs from patients with melanoma can prevent tumor relapse by downregulating β-catenin and blocking tumor-cell proliferation in an miR-34a-dependent manner 232. In hepatoma cells, insulin-like growth factor 1 (IGF1) secretion prevents the intercellular exosomal transfer of miR-122, thus promoting the proliferation of neighboring cells by suppressing the expression of miR-122 233. These findings suggest a link between the loss of tumor-suppressor miRNAs in cancer and exosome secretion.

### saRNAs

Small activating RNAs (saRNAs) are small dsRNAs of approximately 21 nucleotides. After generating active Ago-RNA complexes, they trigger gene expression activation by targeting promoter regions. In addition, they have an effect in any genomic region with antisense transcripts 234, 235. SaRNAs have received much attention in the field of cancer therapy, as they can enhance the transcriptional activation of tumor-suppressor genes such as p21 236, 237, Wt-1 238, E-cadherin 239, NKX3-1 240, and PTEN 241 through various mechanisms 236, 242, 243, by inducing cell-cycle arrest, inhibiting proliferation, inducing apoptosis, inhibiting metastasis, and reversing multidrug resistance 236, 242, 243. Chemical modification could overcome the endonuclease resistance, serum stability, and off-target effects of saRNAs and nano-delivery systems.

Lipid-based delivery systems are commonly used for systemic or local saRNA delivery. Take target gene P21 as example, results illustrated that using targeting saRNA transcriptionally activated p21 expression and promoted cell-cycle arrest in the G1/G0 phase, inhibiting cell proliferation and enhancing chemosensitivity 236. Local administration of saRNAs has minimal off-target potential. Regarding the therapeutic potential of saRNAs targeting p21, intravesicular delivery of LNP/dsP21-322 results in approximately 40% tumor regression and prolongs survival in a mouse orthotopic model of bladder cancer 244.

Surface modification, for example, with biodegradable polymers such as PEG, can overcome the toxicity and off-target effects of LNP delivery systems 244. Indeed, 2'-fluoro modification of the saRNA backbone improves duplex stability in urine 244. To further increase tumor tissue specificity, a selective rectal delivery system has been designed for colorectal cancer. In this system, saRNA-322 is loaded onto an HA covalently anchored anionic lipid shell to accumulate at the lesion site and effectively target CD44-rich tumor cells 245. In another study, to achieve targeted delivery, pancreatic ductal adenocarcinoma (PDAC)-specific adapters have been used to deliver 2'‑fluoropyrimidine-modified saRNA to tumor cells to activate C/EBPα expression 246. Due to their ability to maintain their structural conformation even under physiologically harsh reducing conditions, aptamers exhibit better structural stability, lower toxicity, and lower immunogenicity. Compared to lipid-mediated delivery, high-affinity aptamers better deliver saRNA to tumor nodules, markedly inhibiting tumor growth and significantly reducing the tumor burden in a xenograft model 246.

### RNA aptamers

RNA aptamers are sequences 20-100 nucleotides in length with complex three-dimensional structures that bind to target molecules with high affinity and specificity via non-covalent pocket interactions 247. Aptamers have high structural flexibility, stability, and specificity. In addition, targeted ligands can confer cancer tissue specificity to antitumor drugs 248, 249. RNA aptamers are classified into three functional categories: antagonists that block the interactions of disease-related targets, agonists that activate target receptor function, and those that target moieties that deliver drugs to cancer tissue 250.

Several clinical trials of aptamers for tumor therapy are ongoing. The guanosine-rich AS1411 is the first oligodeoxynucleotide aptamer to be evaluated in phase I and II clinical trials. It suppresses tumor-cell proliferation by interfering with the stability of Bcl-2 mRNA 251, 252. NOX-A12 is an L-type RNA antagonistic aptamer known as Spiegelmer® that targets chemokine (C-X-C motif) ligand 12 (CXCL-12) 253, mobilizes cells from the protective TME, and induces apoptosis and chemosensitization to have an antitumor effect 254. NOX-A12, a mirror-image oligonucleotide with 40 kDa PEG branches, resists nuclease degradation and is immunologically passive 254, 255.

RNA aptamers are used in combination with other therapeutic agents (e.g., siRNAs, microRNAs, and peptides) for targeted delivery. For example, the combination of a PSMA-specific aptamer for prostate cancer with therapeutic oligonucleotides inhibited oncogene activity in PSMA-expressing cells, thereby having an antitumor effect 256. Combining this aptamer with other functional units can achieve multiple biological effects 250, 257, 258. Similarly, in one study, linking a FOXP3-blocking peptide to an aptamer targeting CD28 functionally inactivated Tregs and enhanced the efficacy of cancer immunotherapy 259.

### CRISPR-Cas9 system

**Figure 6:**
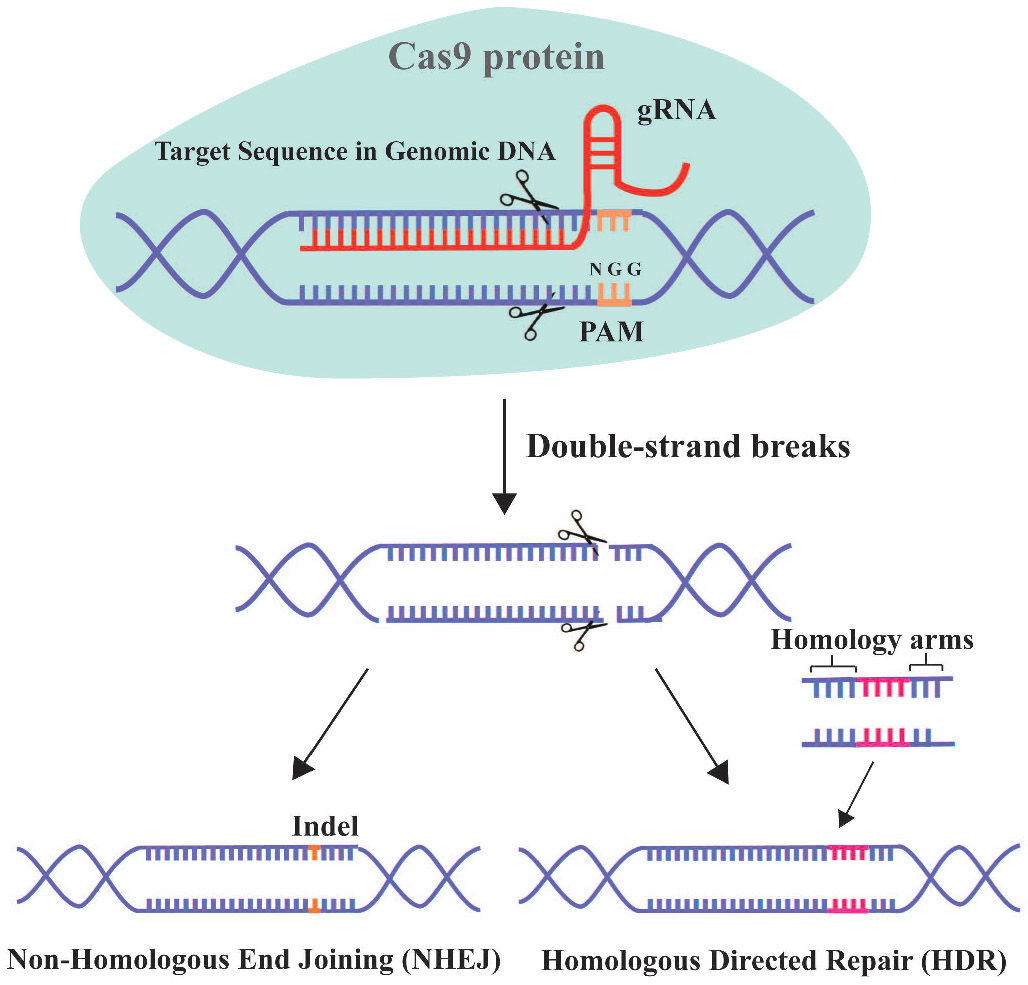
** Schematic diagram of CRISPR-Cas9 technology for gene editing.** Cas9 can specifically target any genomic locus and induce double-strand breaks under the guidance of gRNA. Cells then initiate the repair mechanism by non-homologous end joining (NHEJ) or homologous directed repair (HDR).

The clustered regularly interspaced short palindromic repeat (CRISPR)/Cas9 system has unprecedented therapeutic potential for genetic diseases 16. This adaptive and heritable immune defense system was discovered in bacteria and archaea, and uses short RNAs to guide the degradation of invading viruses, plasmids, or foreign mobile genetic elements 260. CRISPR/Cas9 has progressed since the first report in 2013 of its use for simultaneous precise editing of several sites in the mammalian genome 16, 261. The 2020 Nobel Prize in chemistry was awarded for the discovery of CRISPR RNA (crRNA) and transactivating-crRNA (tracrRNA) 262. The interaction between these two elements forms a two-RNA guide RNA (gRNA), which directs the Cas9 nuclease to the target site (Figure 6).

The two critical components of the CRISPR/Cas9 system are the Cas9 protein and gRNA. The principle of this system involves the insertion of a specific DNA sequence (spacer) from the invading virus and plasmid into the CRISPR locus. Upon re-infection, the CRISPR sites containing the spacer acquired previously are transcribed and the products are processed into mature gRNAs. The gRNA targets Cas9 to a particular genomic locus, where it induces double-strand breaks (DSBs). For binding to and cleavage of DNA by Cas9, the 3'-side of the target sequence must have an NGG protospacer adjacent motif (PAM) 263. The DSB triggers DNA repair by non-homologous end joining (NHEJ) or homologous directed repair (HDR) 264.

Cancer arises from the accumulation of genetic/epigenetic abnormalities. CRISPR-Cas9-mediated genome editing enables precise manipulation of a genomic sequence, enabling the identification of genes involved in carcinogenesis and correction of oncogenic mutations 265, 266. In addition, CRISPR-Cas9 gene editing allows permanent disruption of genes that are essential for tumor survival, potentially circumventing the need for repeated dosing of chemotherapeutics and thereby improving treatment outcomes 267, 268. To selectively kill cancer cells without affecting surrounding normal cells, Kwon* et al.* 269 developed a cancer-specific insertions-deletions (InDels) attacker (CINDELA), which targets cancer-specific CRISPR-mediated DSBs to promote cell death. Notably, CINDELA with CRISPR/Cas9 targets multiple InDels, generating many DNA DSBs. The CINDELA method has been used to kill cancer cells, xenograft cancer cells in mice, patient-derived glioblastomas, and patient-driven xenograft (PDX) lung cancer models without affecting normal cells 269.

However, the CRISPR/Cas9 system has limitations related to cell injury, limited packaging capacity, and immune activation. In addition, the large sizes of Cas9 (160 kDa, 4300 bases) and sgRNA (~31 kDa, 130 bases) preclude the use of viral and nonviral delivery systems 267. The CRISPR-Cas9 gene editing system can be delivered intracellularly using arginine NPs (ArgNPs) to generate SIRP-α knockout macrophages 270. The technique enables tumor penetration and codelivery of the single gRNA and Cas9 to knockdown the “don't-eat-me” signal in macrophages, which prevents engulfment of cancer cells. The technique enhances the phagocytic capacity of macrophages fourfold 270. To address the low editing efficiency and high toxicity of CRISPR-Cas9, Rosenblum* et al.* 267 used lipid NPs to deliver Cas9 mRNAs and sgRNAs. A single intracerebral injection of CRISPR-LNPs for PLK1 (sgPLK1-cLNPs) into an aggressive orthotopic glioblastoma resulted in ~70% gene editing *in vivo*, promoting tumor-cell apoptosis. In addition, the technique allows antibody-targeted delivery for the treatment of diffuse tumors. Intraperitoneal injection of EGFR-targeted sgPLK1-cLNPs resulted in selective uptake into diffuse ovarian tumors, leading to up to ~80% gene editing *in vivo*, inhibiting tumor growth and improving survival 267. A CRISPR/Cas9 codelivery strategy has been used for the treatment of immunological diseases and cancers. In one study, MSNs were encapsulated in lipid layers to form virus-like nanoparticles (VLNs), which protected the RNP complex from enzymatic degradation and prolonged their half-life in the circulation 271. Simultaneous delivery of an RNP complex targeting the PD-L1 gene and the antitumor drug axitinib achieved PD-L1 knockout in cancer cells, significantly reduced immunosuppressive Tregs, and enhanced tumor growth inhibition 271. Zhang* et al.* 272 developed a gene-drug combination that targeted EGFR by specifically inhibiting CRISPR/Cas9 and Sora. Similarly, gene-drug coloaded NPs have been used to stimulate the antitumorigenic pathway in hepatoma carcinoma by inhibiting pro-inflammatory cytokines (IL-6 and IL-8) by regulating the downstream tumorigenic pathway (NF-κB p65) 273. Stimulus-responsive nano systems using NIR-responsive and reducing agent-responsive NPs for codelivery of the Cas9/sgRNA RNP and the antitumor photosensitizer chlorin e6 (ce6) result in the generation of reactive oxygen species (ROS) upon NIR irradiation, facilitating the release of Cas9/sgRNA targeting Nrf2 and enhancing tumor-cell sensitivity to ROS 274. The therapeutic potential of NIR light-triggered systems has been verified by others 275, 276. Although they target different oncogenes, these platforms specifically inhibit the proliferation of cancer cells.

## Perspectives, Challenges, and Conclusion

Although cell and antibody-based therapies currently dominate tumor immunotherapy, more work is needed to overcome the risk for side effects, induction of an inappropriate and potentially harmful immune response, the complexity of treatment, and the high cost of production. RNA-based therapeutics (mRNAs, siRNAs, miRNAs, ASOs, saRNAs, RNA aptamers, and CRISPR gene editing) can modulate the expression of target genes to varying degrees. In addition, they can stimulate an immune response or reshape the suppressive TME by producing antigens or restoring the levels of beneficial proteins. The ability to target multiple genetic components is an advantage of RNA therapeutics over other small-molecule or protein-based drugs. Furthermore, once the chemical structure of the RNA molecule and the *in vivo* delivery system have been developed, RNA-based drugs can be rapidly designed and synthesized. However, several technical bottlenecks affect RNA-based antitumor therapies, including their targeting specificity, safety, and efficacy. To address these issues, a variety of optimization and modification strategies for RNA molecules and delivery systems have been explored.

Therapeutic effectiveness is influenced by off-target effects induced by nonspecific accumulation. To prolong accumulation at tumor sites, promote target uptake, and control drug release, surface modifications with targeting ligands have been developed 277. Target ligand length, density, hydrophobicity, and avidity are determinants of the efficacy of such surface modifications 278. Furthermore, optimization of the particle size, surface charge, and other properties of delivery vehicles would promote selective accumulation of cargo in target organs or tissues. Strategies based on microenvironment-specific targeting release are feasible, including on-demand delivery of CRISPR-Cas9 for precise genome editing. Tumor heterogeneity results in significant variability in the responses—including nonspecific responses—to internal stimuli. The design of next-generation RNA delivery systems should focus not only on reducing the potential toxicity of byproducts but also on overcoming the problems of irreversibility and weak selectivity. Delivery systems will gradually shift from passively responding to actively modulating via several mechanisms, such as regulating tumor-associated immune cells, enhancing tumor cell immunogenicity, and blocking tumor immune-escape mechanisms.

The safety of RNA cargos is a key consideration for their clinical application. The innate immunostimulatory properties of RNA molecules and their function as immune adjuvants enhance the immune response to antigens during vaccine development. However, excessive immunogenicity can lead to severe adverse reactions. Clinical translation requires the striking of a balance between safety and efficacy. The development of more biocompatible delivery vehicles with better biodegradability would accelerate the clinical translation of nanomedicines 279. Novel carriers such as exosomes, bacteriophages, and macrophages could be used to deliver therapeutics such as siRNAs, ASOs, antibodies, and small-molecule drugs. These have considerable potential for gene therapy based on their transport characteristics, prolonged half-life in the circulation, and excellent biocompatibility. Because the components of RNA-based therapeutics are from different cell sources and have different biological functions 280, their safety needs to be systematically evaluated.

Another crucial issue is therapeutic effectiveness. Tumorigenesis involves complex regulatory networks and immune-escape mechanisms. Treating tumors with a single modality is often unsatisfactory. Treatment of tumors requires multiple modalities, potentially including codelivery of cytokines and chemokines, alleviating immunosuppressive signaling in the TME, and *in vivo* targeting of immune cells. Such approaches could enhance the effectiveness of immunotherapy and so reduce the RNA-based drug dose required. Because drugs have different pharmacokinetic and pharmacological characteristics, as well as interindividual differences in drug distribution after systemic administration, multiple-drug treatment strategies must consider the optimal dosage ratio and sequential release to reduce toxicity and enhance efficacy. P-gp inhibitors should be released by delivery systems prior to any co-delivered drugs. Otherwise, the co-delivered drug(s) might be exported extracellularly by P-gp, thus reducing their therapeutic efficacy.

In conclusion, we reviewed the modifications of, delivery systems for, and potential applications of RNA-based therapeutics for cancers. Although RNA delivery platforms have limitations, advances in RNA-based bioengineering mean considerable therapeutic potential for cancer treatment. Indeed, these modalities offer hope for types of cancers with few or no treatment options.

## Figures and Tables

**Table 1:** Overview of clinical trials of RNA-based therapeutics discussed in this review

RNA Types	Name	NCT Number	Phase	Description	Treating diseases	Delivery system
mRNA	CV9201	NCT00923312	I/II	Encoding TAA MAGE-C1, MAGE-C2, NY-SEO-1, survivin, 5 T4	NSCLC	Protamine
	CV9202	NCT03164772	I/II	Encoding TAA: MAGE-C1、NY-ESO-1、MAGE-C2、survivin、5T4, MUC-1	NSCLC	Protamine
	CV9103	NCT00831467	I/II	Encoding TAA: PSA, PSCA, PSMA, STEAP1	Prostate cancer	Protamine
	CV9104	NCT01817738	I/II	Encoding TAA: PSA, PSCA, PSMA, STEAP1, PAP, MUC1	Prostate cancer	Protamine
	BNT111	NCT02410733	I	Encoding TAA: NY-ESO-1, Tyrosinase, MAGE-A3, TPTE	Advanced melanoma	Lipoplex
		NCT04526899	II	Encoding TAA: NY-ESO-1, Tyrosinase, MAGE-A3, TPTE	Advanced melanoma	Lipoplex
	BNT112	NCT04382898	I/II	Encoding TAA: PAP, PSA, three undisclosed antigens	Prostate	Lipoplex
	BNT113	NCT04534205	II	Encoding HPV16 E6 and E7 oncoproteins	Head and neck squamous cell carcinoma	Lipoplex
	BNT116	NCT05142189	I	Encoding NSCLC tumor-associated antigens	NSCLC	Lipoplex
	BNT122	NCT04161755	I	Encoding personalized tumor mutation antigens	Pancreatic	Lipoplex
		NCT03815058	I	Encoding personalized tumor mutation antigens	Advanced melanoma	Lipoplex
		NCT04486378	II	Encoding personalized tumor mutation antigens	Colorectal	Lipoplex
		NCT03289962	I	Encoding personalized tumor mutation antigens	Solid tumors	Lipoplex
	mRNA-4157	NCT03313778	I	Encoding several neoantigens	Solid tumors	LNP
		NCT03897881	II	Encoding 20 different mutated neoepitopes	Melanoma	LNP
	mRNA-5671	NCT03948763	I	Encoding KRAS gene driver mutations (G12C, G12D, G12V, G13C)	NSCLC, pancreatic, colorectal neoplasms	LNP
	CARVac	NCT04503278	I/II	Encoding CLDN6	Solid tumors	Lipoplex
	IVAC	NCT02316457	I	Encoding gp100 3 TAAs selected	TNBC	Lipoplex
	BNT141	NCT04683939	I/II	Encoding IgG antibody	CLDN18.2-positive solid tumors/ solid tumor	Lipoplex
	BNT152+153	NCT04710043	I	Encoding IL-7 and IL12	Solid tumors	Lipoplex
	MEDI1191	NCT03946800	I	Encoding IL-12	Solid tumors	LNP
	mRNA-2416	NCT03323398	I	Encoding OX40L, IL-23, IL-36γ	Solid Tumor Malignancies or Lymphoma; Ovarian Cancer	LNP
	BNT151	NCT04455620	I/II	Encoding IL-2	Solid tumor	Lipoplex
siRNA	ALN-VSP02	NCT01158079; NCT00882180	I	Target ACSL4 and PLK1	Solid Tumors	LNP
	CALAA-01	NCT00689065	I	Target Fibronectin	Solid Tumor	cyclodextrin polymer
	TKM-080301	NCT01437007; NCT02191878; NCT01262235	I/II	Target TGFBR2	Colorectal Cancer with Hepatic Metastases Pancreas Cancer with Hepatic Metastases Gastric Cancer with Hepatic Metastases	LNP
	siG12D LODER	NCT01676259; NCT01188785	II	Target K-ras	Pancreatic Ductal Adenocarcinoma Pancreatic Cancer	Polymers
	iExosomes	NCT03608631	I	Target K-ras	Metastatic Pancreatic Adenocarcinoma Pancreatic Ductal Adenocarcinoma	Exosomes
	Atu027	NCT01808638; NCT00938574	I/II	Target 4E-BP1	Carcinoma, Pancreatic Ductal Advanced Solid Tumors	LNP
	DCR-MYC	NCT02314052; NCT02110563	I/II	Target MYC	Hepatocellular Carcinoma/ Solid Tumors	LNP
saRNA	MTL-CEBPA	NCT05097911; NCT02716012; NCT04105335	I	Target CCAAT/enhancer binding protein alpha (C/EBP-α).	Hepatocellular Carcinoma Liver Cancer	
miRNA	MRX34	NCT01829971; NCT02862145;	I/II	Target miR-34	Primary Liver Cancer SCLC Lymphoma Melanoma Multiple Myeloma Renal Cell Carcinoma NSCLC	
	MesomiR-1	NCT02369198	I	Target miR-16	Malignant Pleural Mesothelioma Non-Small Cell Lung Cancer	
Aptamer	AS1411	NCT00512083; NCT00881244; NCT01034410	I/II	Target nucleolin	Leukemia, Myeloid/solid tumors	
	NOX-A12	NCT03168139; NCT00976378; NCT01194934	I/II	Target CXCL-12	Metastatic Colorectal Cancer/ Metastatic Pancreatic Cancer/ Hematopoietic Stem Cell Transplantation	

**Table 2:** Nanoplatform for delivery of siRNA in anti-tumor therapy

Types	Nanocarriers	Function of the nanocarriers	Targeted gene	Treating diseases	Injection methods	Ref
Targeting	Cationic lipid nanoparticles	Enhance tumor-targeted delivery	CD47	Triple negative breast cancer; Melanoma	Intravenous injection	210
	Apolipoprotein E3-reconstituted high-density lipoprotein with a CaP	Enhance the Ras-activated cancer cells to swallow drugs	ATF5	Glioblastoma	Intravenous injection	281
	Hybrid nanocomplex	Target CD44 on TNBC cells; higher cellular uptake and better tumor penetration of the encapsulated cargos	IKBKE	Triple-negative breast cancer	Peritumoral injection	198
	Tumor-targeted lipid-dendrimer-calcium-phosphate nanoparticles	Enhanced gene delivery capacity and immune adjuvant properties by activating the STING-cGAS pathway	PD-1	Hepatocellular carcinoma	Intravenous injection	200
	Nanoliposomes	Delivery to target cells and affect tumor cells and infiltrating lymphocytes	PD-1	Melanoma	Intravenous injection	203
Stimulus responsiveness	CaP-phospholipid complex nano delivery system	PH sensitivity; Protects siRNA by endogenous RNases	As2O3; HER2	Gastric cancer	Intravenous injection	194
	Liposomal nanocarrier	PH-sensitive	Bcl-2	Melanoma	Intravenous injection	197
	Nucleic acid nanogel	Kill tumor cells photodynamically and induce remarkable immunogenic cell death	PD-1	Melanoma	Intravenous injection	208
	Photolabile spherical nucleic acid	NIR-sensitive, designable and biocompatible merits	HIF-1α; Bcl-2	Cervical cancer	Intravenous injection	282
Surface chemistry	Lipidoid nanoparticles	Facilitate interaction with the cell membrane; Endosomal escape in other tissues	HoxA1	Premalignant breast lesion	Direct nipple injection	192
	Cationic nanoparticles	Prolong RNA circulation and augment cell uptake	SMO	Glioma; medulloblastoma	Intravenous injection	193
	Solid lipid nanoparticles	Engineered with lecithin and cholesterol and were surface-modified with acid-sensitive sheddable PEG	PD-1	Melanoma	Intratumoral injection	206
	Stable nucleic acid-lipid nanoparticle	Enable high encapsulation Efficiency of nucleic acids with improved cellular uptake and subsequent release	CD47	Colon cancer	Intravenous injection	212
	Neutral nano-liposomal carrier	Process a high rate of in vivo tumor reducing capability	mTOR	Mammary carcinogenesis	Intravenous injection	207
